# Low Temperature (Down to 6 K) and Quantum Transport Characteristics of Stacked Nanosheet Transistors with a High-K/Metal Gate-Last Process

**DOI:** 10.3390/nano14110916

**Published:** 2024-05-23

**Authors:** Xiaohui Zhu, Lei Cao, Guilei Wang, Huaxiang Yin

**Affiliations:** 1 Integrated Circuit Advanced Process R&D Center, Institute of Microelectronics, Chinese Academy of Sciences, Beijing 100029, China; zhuxiaohui@ime.ac.cn (X.Z.); caolei@ime.ac.cn (L.C.); 2 School of Integrated Circuits, University of Chinese Academy of Sciences, Beijing 100049, China; 3 Key Laboratory of Fabrication Technologies for Integrated Circuits, Institute of Microelectronics, Chinese Academy of Sciences, Beijing 100029, China; 4 Hefei National Laboratory, University of Science and Technology of China, Hefei 230088, China; wangguilei@hfnl.cn

**Keywords:** SOI stacked nanosheet, cryo-CMOS, quantum transport, fishbone FET

## Abstract

Silicon qubits based on specific SOI FinFETs and nanowire (NW) transistors have demonstrated promising quantum properties and the potential application of advanced Si CMOS devices for future quantum computing. In this paper, for the first time, the quantum transport characteristics for the next-generation transistor structure of a stack nanosheet (NS) FET and the innovative structure of a fishbone FET are explored. Clear structures are observed by TEM, and their low-temperature characteristics are also measured down to 6 K. Consistent with theoretical predictions, greatly enhanced switching behavior characterized by the reduction of off-state leakage current by one order of magnitude at 6 K and a linear decrease in the threshold voltage with decreasing temperature is observed. A quantum ballistic transport, particularly notable at shorter gate lengths and lower temperatures, is also observed, as well as an additional bias of about 1.3 mV at zero bias due to the asymmetric barrier. Additionally, fishbone FETs, produced by the incomplete nanosheet release in NSFETs, exhibit similar electrical characteristics but with degraded quantum transport due to additional SiGe channels. These can be improved by adjusting the ratio of the channel cross-sectional areas to match the dielectric constants.

## 1. Introduction

At present, constructing high-performance semiconductor quantum devices with advanced complementary metal oxide semiconductor (CMOS) technology is one of critical methods for developing modern quantum computing systems. There are a series of developed silicon qubits based on specific SOI planar, fin and nanowire (NW) field-effect transistors (FETs) that have achieved multi-quantum dot characteristics and high-fidelity gate operation behaviors based on general single-quantum theory [1,2,3]. These results suggest the feasibility of Si quantum dot devices developed for advanced Si MOS devices or processes.

With the continuous development of Moore’s Law and the scaling of CMOS technology nodes, MOS transistor structures are continuing to evolve [4,5]. Si FinFETs with a high-k/metal gate-last (HK/MG-Last) process is a mainstream CMOS manufacturing technology currently being mass-fabricated [6,7]. Nanosheet (NS) FETs exhibit better gate control, higher integration density and good process compatibility with current mass-manufacturing processes, and they are considered to be the next-generation mainstream MOSFET structure beyond FinFETs [8,9,10]. Exploring the implementation path of Si quantum devices by utilizing the low-temperature quantum properties of more advanced Si MOS devices can not only break through the integration scale challenges of traditional Si quantum devices with specific structures, but this can also promote the emergence of quantum computing with new possibilities in the future.

Due to the greater gate-field control of stacked ultra-thin Si channels, their quantum constraints are expected be more pronounced. Some first-principles and density functional theory (DFT) computational methods have been utilized to calculate the electrical properties of stacked NSs and have been employed to guide the design of stacked NS devices [11,12]. Unfortunately, although there have been some studies on the low-temperature characteristics of NS transistors for specific channel structures and customized processes [13,14,15,16,17,18,19], there is little research on the low-temperature carrier transport characteristics of NS transistors with a general structure using mainstream fabrication processes based on HK/MG-last technologies. Furthermore, data on the quantum transport characteristics and relative quantum dot operation behavior of NS devices are also lacking.

In this paper, based on the developed SOI stacked nanosheet (NS) transistors with a HK/MG-last process, the electrical and quantum transport characteristic of NS transistors are systematically investigated at ultra-low temperatures (down to 6 K). The differences in these characteristics under different process and structural conditions were also compared, which are well explained with hybrid simulation methods.

## 2. Materials and Methods

The stacked nanosheet (NS) transistors are fabricated on the 8-inch CMOS pilot line of the Institute of Microelectronics, Chinese Academy of Sciences, and the developed fabrication process is highly compatible with those adopted for future mass-manufacturing processes [10,20]. The transistors are fabricated on an 8-inch SOI wafer with a diameter of 200 mm. Firstly, the top silicon thickness of the SOI substrate is reduced to 40 nm using the sacrificial oxidation method. Subsequently, three-layer SiGe/Si superlattice structures with a thickness of 12 nm/18 nm were grown on it using epitaxy technology. A fin is formed using sidewall transfer technology and the stacked portion is exposed, while a dummy ploy-Si gate stack is patterned by electron beam and reactive ion etching. The self-aligned source–drain is formed by using a silicon nitride spacer and HDD ion implantation (BF+, dose of 5 × 10^20^ and energy of 5 keV) along with an RTA at 850 °C for 30 s. Due to the absence of traditional LDD injection, a natural bottom-doping region is formed in the doping region, which may form a potential barrier under specific conditions.

After a poly open polish (POP) process and dummy gate-stack removal, the SiGe layers in the channel are highly selectively etched from the fin with a mixture of corrosion solutions, which finally cause the formation of stacked Si NS channels. Another innovated method has been developed in this process flow. With the partial etching of the SiGe layers in the channel, a special fishbone transistor structure was also developed for better NS device performance, which has been mentioned in our previous work [21]. After the nanosheet channel release, the multi-layered HK/MG structure was formed via the atomic layer deposition (ALD) process. Finally, the metal contact process for interconnection was performed.

**Figure 1:**
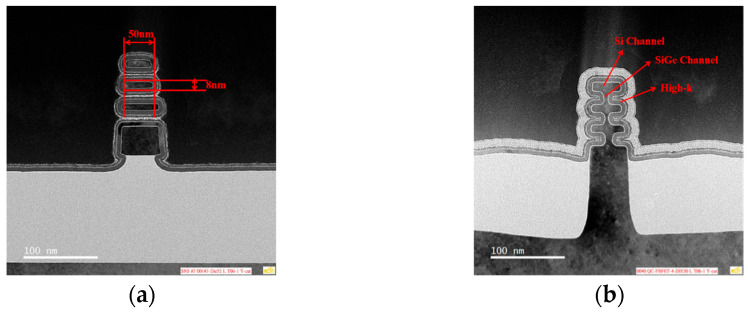
Cross-sectional TEM of the SOI stacked NS transistor with HK/MG process. (**a**) Ordinary SOI stacked NS transistor; (**b**) Fishbone transistor.

The device structures were observed by dark-field transmission electron microscopy (TEM), and the results obtained are depicted in Figure 1. Clear observation of triple-stacked channels is achieved in the stacked NS, while in the fishbone FET, residual SiGe channels are observed in addition to the stacked channels. The structural parameters of the stacked nanosheets and the distinct channels in the fishbone FET are also annotated. The electrical characteristics of this SOI stacked NS FET device were measured in the vacuum chamber at temperatures ranging from 6 K to 300 K with the Lake Shore CRX-4K system and 4200-SCS semiconductor parameter analyzer (Lake Shore Cryotronics, Westerville, OH, USA).

## 3. Results

### 3.1. Low-Temperature Electrical Characteristics of SOI Stacked NS FET

**Figure 2:**
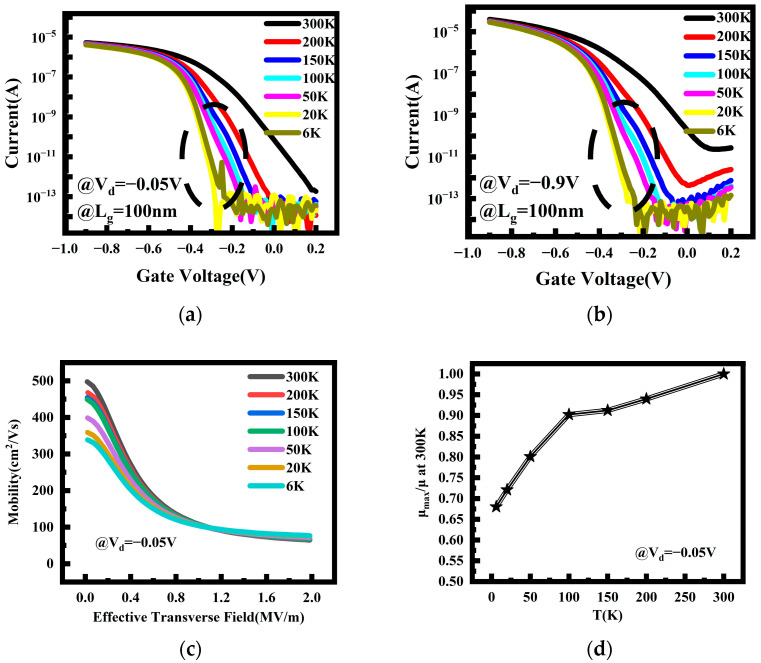
Electrical characteristics of a P-type SOI stacked NS FET with a gate length of 100 nm; (**a**) *V_d_* = −0.05 V; (**b**) *V_d_* = −0.9 V; (**c**) Low-field carrier mobility vs. the effective transverse field at *V_d_* = −0.05 V; (**d**) The maximum value of the mobility vs. temperature at *V_d_* = −0.05 V.

The transfer characteristics curves of a P-type SOI stacked NS FET with a gate length of 100 nm are depicted in Figure 2. Figure 2a,b corresponds to the device operation in the linear region (*V_d_* = −0.05 V) and saturation region (*V_d_* = −0.9 V), respectively. Both scenarios exhibit typical transistor current characteristics. Figure 2c displays the low-field mobility at *V_d_* = −0.05 V, revealing a rapid decrease in the low-field mobility with an increasing effective transverse electric field, along with the observation of a saturation electric field of 1.2–1.6 MV/m. The relationship between the maximum mobility and temperature is plotted in (d). Due to the higher doping concentration in the channel, the freeze-out effect of carriers predominates with regard to mobility, resulting in a decrease in the maximum mobility with decreasing temperature. The reduction in mobility is relatively minor at temperatures above 100 K. However, as the temperature drops below 100 K, the freeze-out effect of carriers intensifies, leading to a rapid decline in mobility.

As the temperature decreases from 300 K to 6 K, there is a noticeable decrease in the current within the device. In contrast, the off-state current decreases rapidly as the temperature decreases to 50 K. This is primarily due to the corresponding increase in the band-gap width of silicon material and the disappearance of some impurity energy levels as the temperature decreases. This implies the gradual closure of some originally open conduction channels. While the on-state current also decreases, its reduction rate is relatively slower. The main influencing factor is the rapid decrease in carrier concentration within the channel. This difference results in a rapid increase in the device’s on–off ratio, which is the ratio of the on-state current to the off-state current, with decreasing temperature.

**Figure 3:**
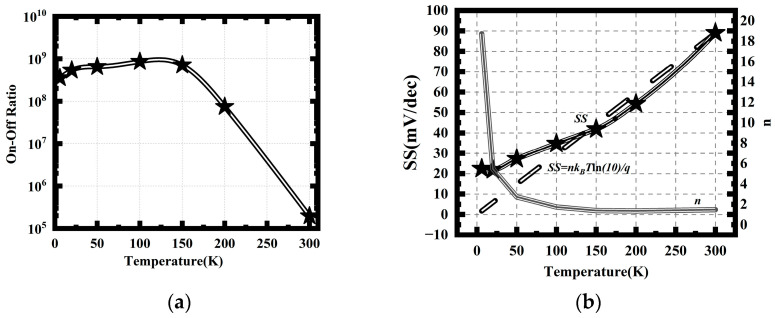
Temperature dependence of CMOS parameters; (**a**) On–off ratio; (**b**) SS.

The on–off ratio increases rapidly with decreasing temperature above 150 K, ranging from 10^5^ at 300 K to 10^9^ at 150 K, as shown in Figure 3a. When the temperature drops below 100 K, the change in the off-state current with temperature becomes weaker, which is consistent with the best characteristics of CMOS mentioned in previous literature at liquid-nitrogen temperature. Most conduction channels brought about by defects and impurity energy levels have already closed, which maximizes the on–off ratio. For temperatures below 100 K, the on–off ratio remains essentially constant. This is because both the off-state and on-state currents weaken in response to temperature, resulting in little variation in the on–off ratio.

Due to the improvement in subthreshold characteristics, the SS of the SOI stacked NS FET decreases with decreasing temperature that is above approximately 50 K, as shown in Figure 3b. At 300 K, the device’s *SS* is around 90 mV/dec, but at 50 K, it drops below 30 mV/dec, significantly below the physical limit at room temperature. In fact, SS exhibits a linear relationship with temperature, which can be expressed by the following formula [22]:

(1) $$SS={nk}_{B}T\frac{ln(10)}{q}$$

The coefficient *n* is related to the device interface defect charges, and for this device, *n* is 1.5 at 300 K. When the temperature decreases to 100 K, the variation in *n* is small, and the relationship between *SS* and temperature also exhibits a basically linear relationship. However, when the temperature drops to 50 K and below, attributed to enhanced interface state effects, the value of *n* starts to increase rapidly, and *SS* also saturates at about 20 K. It has been reported in previous literature that optimizing the interface can improve the *SS* [15]. The minimum value of the *SS* is approximately 22 mV/dec in this device, which is consistent with the findings obtained from testing traditional bulk silicon NS devices [19].

**Figure 4:**
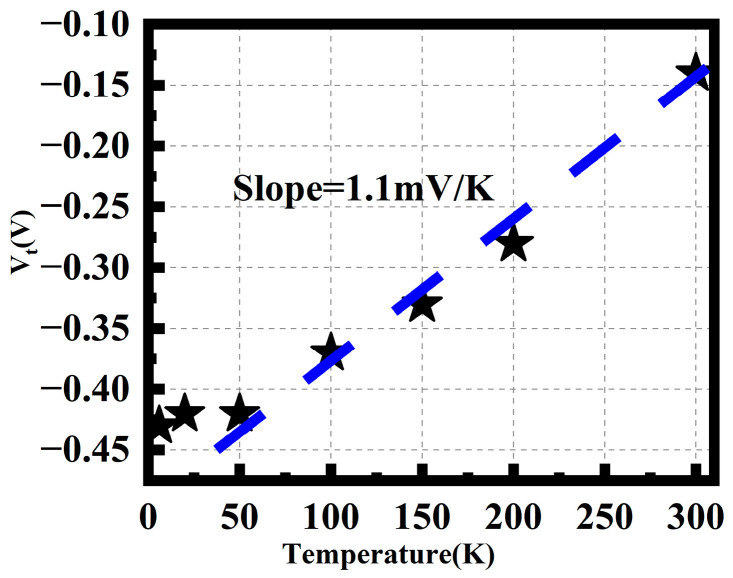
Temperature dependence of the threshold voltage. Blue Line: Linear Fitting.

Threshold voltage is another crucial parameter for transistors. As the temperature decreases, the threshold voltage becomes more negative, as shown in Figure 4. Above 50 K, the threshold voltage exhibits a linear relationship with temperature, with a slope of approximately 1.1 mV/K. The temperature dependence of the threshold voltage for p-type MOSFETs can be expressed by the following formula [23]:

(2) $$\frac{dV_{t}}{dT}=\frac{d\Phi_{F}}{dT}\approx 8.63\times 10^{-5}\left[\ln N_{d}-38.2-\frac{3}{2}\left(1+\ln T\right)\right]$$

The temperature coefficient slope values calculated for this device range from 0.84 to 1.31 mV/K within the temperature range of 50–300 K. These calculations validate the reasonableness of the fitted value of 1.1 mV/K. The temperature variation of the threshold voltage is primarily associated with the temperature change of the material’s band gap. At extremely low temperatures, the band-gap width of silicon material increases, resulting in an increase in the threshold voltage. The saturation of the threshold voltage increase may be attributed to the influence of defect levels. Additionally, some significant quantum effects also begin to play a role, further weakening the temperature dependence of the threshold voltage, leading to saturation of the threshold voltage at extremely low temperatures.

Overall, at low temperatures down to 6 K, the improved mobility of carriers within the crystal enhances the on-state current, while the closure of some leakage paths due to partial freezing results in a significant reduction in the off-state current. Consequently, this enhances the device’s switching speed and characteristics. Simultaneously, the *SS* also decreases rapidly. Therefore, SOI stacked NS FET devices exhibit superior performance at low temperatures, potentially providing advantages for low-power electronic devices and high-performance computing.

### 3.2. Quantum Transport at Low Temperature for SOI Stacked NS FETs

**Figure 5:**
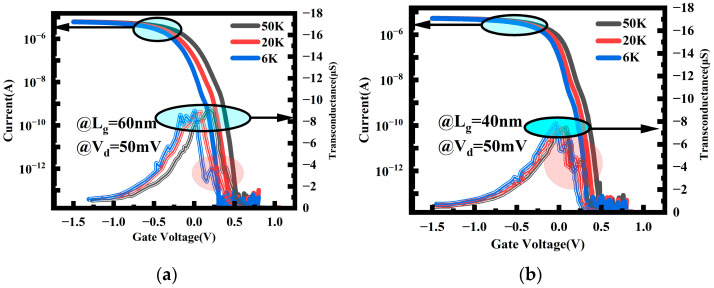
Ballistic transport characteristics of 60 nm (**a**) and 40 nm (**b**) gate-length devices below 50 K.

The transfer characteristics curves shown in Figure 2 reveal a unique current behavior, displaying step-like currents induced by quantum effects. This current characteristic is more pronounced in devices with shorter gate lengths. To further understand this phenomenon, the current characteristics of devices with gate lengths of 40 nm and 60 nm below 50 K are plotted in Figure 5, where the step-like currents generated by quantum effects can be clearly observed. The transconductance in two scenarios is also plotted in Figure 5, clearly demonstrating non-smooth transconductance peaks (highlighted in the red circles) resulting from current steps outside the main peak. This current characteristic is particularly significant for devices with shorter gate lengths (40 nm) and lower temperatures (6 K), indicating a quantum ballistic transport result.

**Figure 6:**
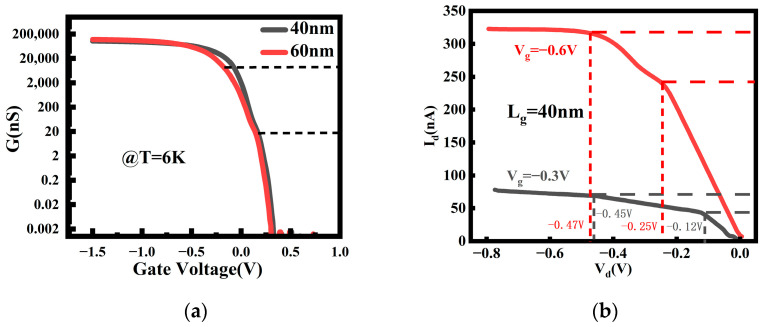
(**a**) The channel conductance as a function of gate voltage at a temperature of 6 K; (**b**) *I_d_*–*V_d_* graph for the device with a channel length of 40 nm.

The channel conductance as a function of gate voltage at a temperature of 6 K was plotted in Figure 6, along with the *I_d_*–*V_d_* graph for the device with a channel length of 40 nm. The current steps induced by quantum transport are observed to be more pronounced. The *I_d_*–*V_d_* characteristic also exhibits a step-like behavior in Figure 6b, with different slopes of the channel current at different voltage regimes. This is because, as *V_d_* changes, the drain’s Fermi level also varies. When the Fermi level crosses the bottom of the sub-band available for current conduction, new current transport modes emerge. Consequently, the slope of the current–voltage curve exhibits a discontinuity at this point. Due to the adoption of ultra-thin NS channels, compared with larger-sized nanowire and nanosheet channels [24,25], the quantum transport effect is more prominent, allowing for the more pronounced current steps resulting from ballistic transport to be observed.

To further understand this phenomenon, the Wharam formula can be introduced to calculate channel conductance [26]:

(3) $$G=\frac{2e^{2}}{h}\sum_{i}T_{i}$$

where $T_{i}$ is the transmission coefficient of the i sub-band. This formula explicitly reveals the relationship between the conductivity and the transmission coefficients of each sub-band. For conductivity, as the gate voltage increases, new sub-bands are triggered to participate in current transport, leading to a significant change in channel conductivity. When the gate voltage is low, only the lowest energy level sub-band participates in transport, resulting in relatively low channel conductivity. However, as the gate voltage increases, more sub-bands are excited and participate in current transport, causing the conductivity to gradually increase, eventually exhibiting the observed step-like conductivity characteristic in Figure 6a.

Under fixed gate voltage conditions, the current variation in relation to the source–drain voltage can be described by a simple formula:

(4) $$I_{d}=M\frac{2e^{2}}{h}V_{d}$$

where M is the number of sub-bands involved in current transport. A higher gate voltage implies the involvement of more sub-bands in transport, indicating a larger value of current, thereby resulting in a significant increase in the current. The value of *M* in this experiment can be calculated to be between 5 and 21, which slightly exceeds the typical range for one-dimensional transport (between 1 and 10) and demonstrating that the device exhibits non-typical partial one-dimensional transport characteristics. As *V_d_* changes, the drain’s Fermi level also varies. When the Fermi level crosses the bottom of the sub-band available for current conduction, new current transport modes emerge. Consequently, the slope of the current–voltage curve undergoes a discontinuity at this point.

In Figure 6b, it is observed that the height of the first step (about 43.35 nA for V_g_ = −0.3 V and 242.93 nA for V_g_ = −0.6 V) is approximately twice the height of the second step (about 23.51 nA for V_g_ = −0.3 V and 103.28 nA for V_g_ = −0.6 V). This is determined by the band structure of silicon. The first sub-band of the silicon conduction band has a doubly degenerate bottom, meaning that two energy levels at the bottom of the conduction band have the same energy. As the voltage increases, both of these degenerate levels must be filled simultaneously for electrons to enter the next transport sub-band. This is reflected in the current and conductivity plots as the height of the first step is approximately twice the height of the second step.

**Figure 7:**
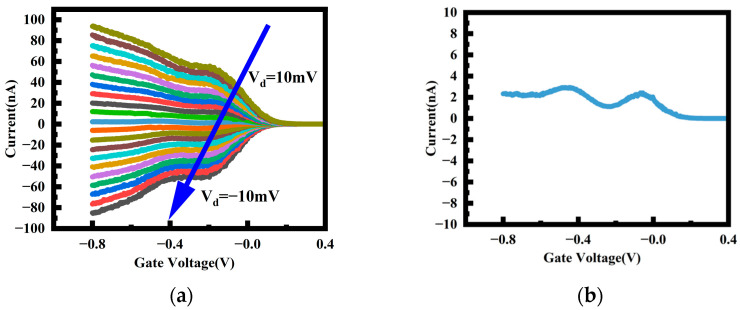
(**a**) I_d_–V_g_ of a device with L_g_ = 40 nm at 6 K temperature (The voltage V_d_ varies from 10 mV to −10 mV along the direction of the blue arrow); (**b**) Current at 0 V.

Furthermore, the device with a gate length of 40 nm was measured under low bias conditions to investigate its transport characteristics over a larger range in Figure 7a, revealing distinct step-like changes in the current with variations in the gate and drain voltages. This step-like current phenomenon directly reflects partial one-dimensional quantum transport. Notably, the situation is largely symmetric for both positive and negative values of *V_d_*. However, Figure 7b displays the current at *V_d_* = 0 V, revealing that there is still a non-zero current present when the source–drain voltage is 0. This phenomenon has also been observed in other literature; for instance, in circular nanowires [23]. It arises from the asymmetric triangular potential barrier between the source and drain [27]. For a triangular potential barrier, the tunneling probabilities in the two directions are different. In this case, the triangular barrier provides a potential difference of about 1.3 mV. When the difference in tunneling probabilities on the two sides of the trapezoidal or triangular potential barrier is significant, this current can become quite substantial.

### 3.3. Comparison of Quantum Properties between NS and Fishbone Transistors

The structure of a fishbone FET is illustrated in Figure 1b. In a fishbone FET, there exists deliberately designed SiGe fin channels between stacked Si NS channels for the developed partial removal process of SiGe [21]. The proposed fishbone FET not only exhibits a significantly enhanced driving current but also provides a good balance between the performances of n-type and p-type Fishbone FETs with little extra process cost.

The characteristics of the fishbone FET at low temperatures are depicted in Figure 7a. The current of NS devices with the same gate length at 6 K is also plotted in the figure for comparison. For the fishbone FET, its overall current trend is like that of the NS device with the same gate length. By utilizing the constant current method to extract the threshold voltage at a current of $1\times 10^{-8}$ A, a variation of the threshold voltage with temperature is obtained, as shown in Figure 7b. It can be observed that the threshold voltage of the fishbone FET is shifted further left compared with the NS device. This is because the gate is no longer fully surrounding, which results in weakened gate control over the channel that require higher voltages for device activation, thus shifting the threshold voltage to the left. As the temperature decreases, the threshold voltage of the fishbone FET slightly decreases. When the temperature decreases from 50 K to 6 K, the threshold voltage changes from −0.31 V to −0.42 V. In contrast, the threshold voltage of the NS FET changes from 0.26 V to 0.03 V. The temperature response of the fishbone FET is weaker compared with that of the NS FET, as the presence of the SiGe channel introduces additional interfaces and interface states. These additional interface states partially offset the threshold voltage shift caused by the temperature reduction.

**Figure 8:**
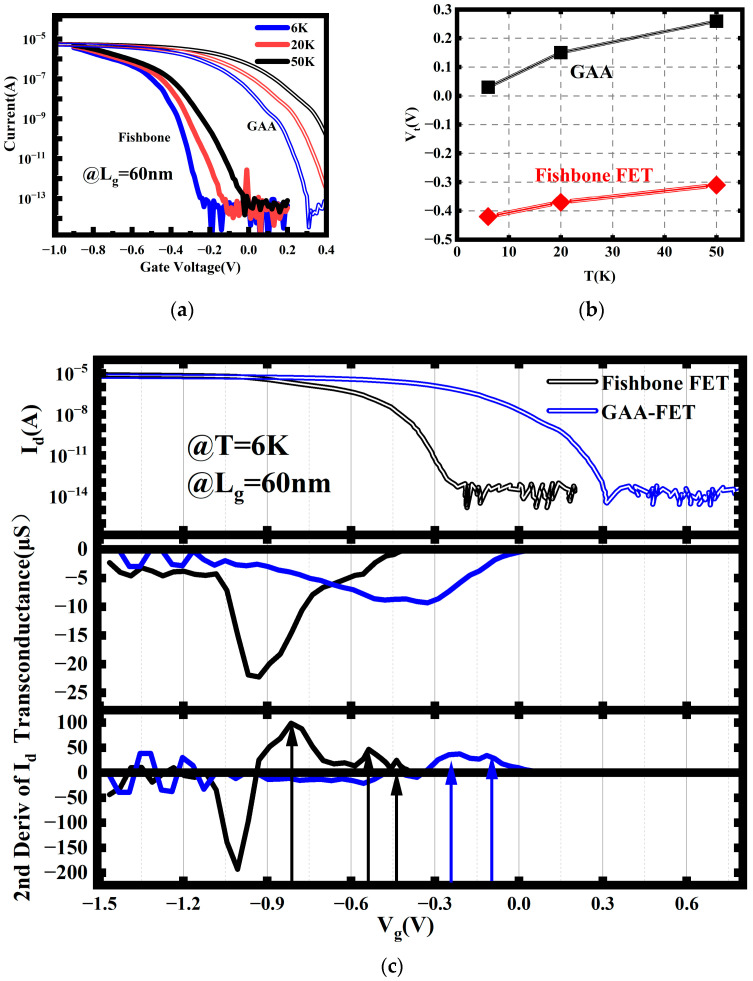
(**a**) I_d_–V_g_ characteristics and (**b**) threshold voltage of the fishbone FET. (**c**) Current and transconductance and the second-order derivative of the current of the fishbone FET and the NS at 6 K. Arrows: The peak of the 2nd derivative of the current.

Further observation of the current curves reveals that for the fishbone FET, there exists a current staircase region like that of the stacked NS FET, but the variation within the current staircase region is more complex compared with that of the stacked NS FET. To further investigate the current of the fishbone FET at 6 K and to better visualize its changes, the transconductance values were calculated. To provide a clearer representation of the transconductance variation, the derivative of the transconductance with respect to gate voltage, i.e., the second derivative of the current with respect to gate voltage, is also plotted in Figure 8c.

The current and transconductance of both the fishbone FET and the stacked NS FET exhibit similar characteristics; both currents gradually increase and tend to saturate at higher gate voltages, while the transconductance shows a prominent peak. However, their shapes differ; the transconductance peak of the stacked NS FET appears as a relatively flat and broad peak, whereas that of the fishbone FET is a narrower and sharper peak. This is because, for the stacked NS FET, strong electric fields exist at the four corners, causing them to open first. Then, as the gate voltage increases, other parts of the channel gradually open, resulting in a relatively long process and hence a broad, flat transconductance peak. In contrast, the presence of additional SiGe/Si interfaces and SiGe channels in the fishbone FET, along with more corners, leads to faster inversion at the corners due to the strong electric fields. As a result, the current quickly saturates after transistor turn-on, leading to a sharper transconductance peak.

The stacked NS FET, due to its high symmetry, exhibits fewer artifacts in its transconductance peak. The image presents typical features of partial one-dimensional ballistic transport. However, in contrast, the transconductance of the fishbone FET displays richer fine structures than the stacked NS FET, which is attributable to the disruption of vertical device symmetry by the additional SiGe channel. The intricate structure of the transconductance peak arises from the superposition of currents from various channels, such as the main channel and the vertical channels. This results in more peaks in the second derivative of the current and even finer structures between the two peaks in the fishbone FET.

**Figure 9:**
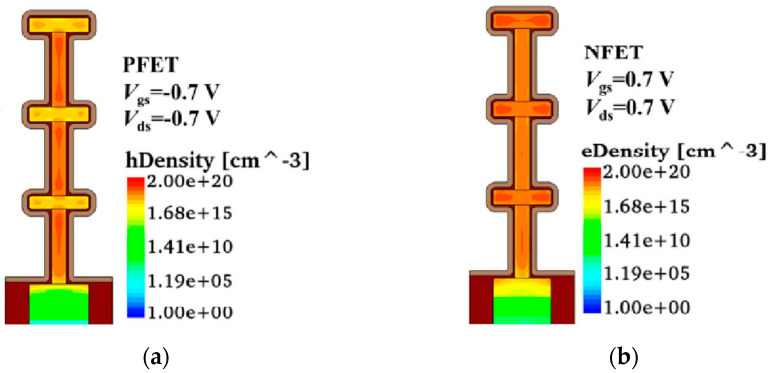
The carrier distribution of the fishbone FET. (**a**) PFET; (**b**) NFET.

The electrical conductance characteristics were investigated with TCAD simulation tools. The simulation was conducted using Sentaurus TCAD 2017, developed by Synopsys. The structural parameters of the fishbone FET were set as follows. The width of the silicon nanosheet was 50 nm, and the height was 8 nm. The width of the SiGe channel was 8 nm, and the height was 75 nm. To qualitatively illustrate the distribution of carriers in the vertical channel, the height of the SiGe layer was set higher than in the experiments to make the carrier distribution clearer. It is evident that besides the main channel of the Si nanowire, the vertical SiGe channel also contributes to current transport. The carrier distribution maps in Figure 9 clearly show the carrier accumulation in the SiGe channel.

**Figure 10:**
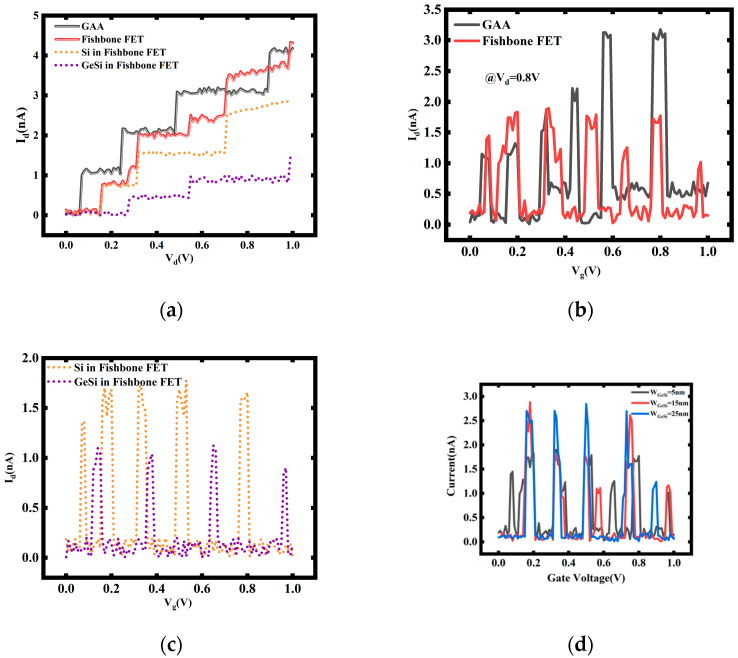
The current obtained through the master equation model simulation includes the following: (**a**) *I_d_*–*V_d_* for W_SiGe_ = 5 nm; (**b**) *I_d_*–*V_g_* for W_SiGe_ = 5 nm; (**c**) the contribution of Si and SiGe channels to *I_d_*–*V_g_* for W_SiGe_ = 5 nm; (**d**) *I_d_*–*V_g_* for different W_SiGe_ values.

Another simulation was also conducted. In this case, the nanosheet width and height were set to 50 nm and 8 nm, respectively, and the height of the SiGe channel was set to 12 nm. The width of the SiGe channel, W_SiGe_, was set to 5 nm. Two 2 nm thick SiO_2_ layers were placed as potential barriers between the source–drain and the channel of the device, respectively. The stacked NS FET with the same nanosheet structural parameters was also included in the calculation. After performing simple TCAD simulations, we extracted the band structure and carrier distribution parameters from the results. Treating the device as a quantum dot, we utilized the Wentzel–Kramers–Brillouin (WKB) theory to calculate the tunneling capacitance and tunneling resistance. Subsequently, these parameters were incorporated into the quantum transport equations to obtain the quantum characteristics of the device [28,29,30,31]. The results are presented in Figure 10.

The contributions of quantum current from the SiGe channel are evident from the dashed lines in Figure 10a,c. Due to the differing band structures of the Si main channel and the vertical SiGe channel, the positions of the current steps and conductance peaks resulting from quantum effects are also different. The superposition of these effects makes the current behavior of the fishbone transistor more complex compared with the stacked NS FET.

Furthermore, simulations are conducted with different widths of the SiGe channel, as shown in Figure 10d. It is observed that when the SiGe channel width W_SiGe_ = 25 nm, the current peaks in the *I_d_*–*V_g_* curve do not exhibit significant additional peaks, unlike the other two cases, where the peaks appear more scattered.

This is because with a SiGe channel width of 25 nm, the cross-sectional area of the SiGe channel is 300 nm^2^, while the cross-sectional area of the Si channel is 400 nm^2^. The ratio between them is 1.33, which is close to the ratio between the dielectric constants of SiGe and silicon (1.0~1.36). Therefore, their tunneling capacitance values are also similar. As tunneling capacitance couples with the source–drain, it implies that the overall quantum characteristics of the two channels are similar, resulting in improved mixed current characteristics when combined. This suggests that adjusting the ratio of the cross-sectional areas of channels made of different materials to match the ratio of their dielectric constants can match their quantum characteristics, thus obtaining better device performance.

## 4. Conclusions

In summary, this study experimentally investigated the low-temperature electrical and quantum characteristics of SOI stacked NS and innovated fishbone transistors. The NS devices exhibit much improved switching characteristics, especially in the off-state leakage current at ultra-low temperatures (6 K). Additionally, the threshold voltage demonstrates a linear decrease with decreasing temperature above 50 K, exhibiting excellent agreement with theoretical predictions. A quantum ballistic transport characteristic is also observed in the transistor at ultra-low temperatures, particularly evident in shorter gate lengths and lower temperatures. An abrupt change in the slope of the current–voltage relationship occurs that reflects the involvement of new sub-bands in transport with the NS structure. Additionally, a current under zero bias exists, which is thought to be indicative of the presence of the asymmetric triangular potential barrier. The fishbone FETs demonstrate similar electrical behaviors to SOI NS devices but exhibit degraded quantum transport characteristics at ultra-low temperature due to the existence of SiGe fin channels between the Si NSs, which may result in the formation of additional peaks in the transconductance plot that arise from the different quantum effects in channels made of different materials. With a developed hybrid simulation approach by using TCAD and a simplified master equation, the quantum characteristics can be improved by adjusting the ratio of the cross-sectional areas of channels made of different materials to match the ratio of their dielectric constants.

## Data Availability

The data presented in this study are available on request from the corresponding author.
